# Arbitrary Symbolism in Natural Language Revisited: When Word Forms Carry Meaning

**DOI:** 10.1371/journal.pone.0042286

**Published:** 2012-08-06

**Authors:** Jamie Reilly, Chris Westbury, Jacob Kean, Jonathan E. Peelle

**Affiliations:** 1 Department of Speech, Language, and Hearing Sciences, University of Florida, Gainesville, Florida, United States of America; 2 Department of Psychology, University of Alberta, Edmonton, Alberta, Canada; 3 Department of Physical Medicine and Rehabilitation, Indiana University School of Medicine, Indianapolis, Indiana, United States of America; 4 Center for Cognitive Neuroscience and Department of Neurology, University of Pennsylvania, Philadelphia, Pennsylvania, United States of America; Utrecht University, Netherlands

## Abstract

Cognitive science has a rich history of interest in the ways that languages represent abstract and concrete concepts (e.g., *idea* vs. *dog*). Until recently, this focus has centered largely on aspects of word meaning and semantic representation. However, recent corpora analyses have demonstrated that abstract and concrete words are also marked by phonological, orthographic, and morphological differences. These regularities in sound-meaning correspondence potentially allow listeners to infer certain aspects of semantics directly from word form. We investigated this relationship between form and meaning in a series of four experiments. In Experiments 1–2 we examined the role of metalinguistic knowledge in semantic decision by asking participants to make semantic judgments for aurally presented nonwords selectively varied by specific acoustic and phonetic parameters. Participants consistently associated increased word length and diminished wordlikeness with abstract concepts. In Experiment 3, participants completed a semantic decision task (i.e., abstract or concrete) for real words varied by length and concreteness. Participants were more likely to misclassify longer, inflected words (e.g., “apartment”) as abstract and shorter uninflected abstract words (e.g., “fate”) as concrete. In Experiment 4, we used a multiple regression to predict trial level naming data from a large corpus of nouns which revealed significant interaction effects between concreteness and word form. Together these results provide converging evidence for the hypothesis that listeners map sound to meaning through a non-arbitrary process using prior knowledge about statistical regularities in the surface forms of words.

## Introduction

One of the most prominent and longstanding distinctions in cognitive science involves the unique ways that natural languages represent concrete relative to abstract concepts. For most of us, concrete words such as *beach* tend to rapidly evoke a range of perceptual and affective associations (e.g., suntan lotion, salty odor, the sound of crashing waves). In contrast, abstract words such as *condition* and *aspect* typically fail to evoke strong multi-modal perceptual imagery. Psycholinguists have operationally defined word concreteness as the strength with which a word can be experienced directly through the senses; imageability is a strongly correlated construct that describes the strength with which a word can rapidly evoke a mental image [1]–[4]. These definitions for concreteness and imageability have afforded an empirical means for quantifying these semantic constructs comparable to other psycholinguistic variables such as word frequency or word length. To date, researchers have collected subjective concreteness and imageability ratings for many thousands of words spanning grammatical classes (e.g., nouns and verbs) and a wide range of natural languages (e.g., English, Italian, Dutch, Portuguese) in an effort to elucidate one of the most robust phenomena in language, the word concreteness effect.

The word concreteness effect refers to the collective advantage afforded to concrete relative to abstract words across many cognitive domains including age of acquisition, word list memory, spelling accuracy, speed of word recognition, and naming latency [5]–[14]. Young children, for example, typically acquire concrete words long before they demonstrate a similar level of proficiency for abstract words. In addition, this concreteness advantage often resurfaces at the other end of the lifespan as a function of neurological injury. Several forms of stroke aphasia, for example, have been observed to produce selective deficits in either naming abstract words (i.e., abstract word anomia) or in verbal comprehension of abstract words (i.e., abstract word deafness) [7], [15]–[18]. Although less common, the opposite pattern–relatively spared abstract word access compared to concrete word access–has also been documented [19]–[23]. This double dissociation is strong evidence that access to the two word categories is represented in terms of at least a partially dissociable neural representation, a claim which is has been further buttressed by brain imaging evidence [6], [24]–[28]. Thus, converging evidence from a number of distinct sub-disciplines and methodologies lend support to the hypothesis that abstract and concrete words are in many ways unique, both in terms of their unique lexical-semantic properties and their neural representation.

Numerous theories have been posed over the last four centuries to account for the word concreteness effect [29]–[32]. However, a common thread linking theories of word concreteness lies in the lexical-semantic structures of abstract versus concrete concepts. We hypothesize that in addition these semantic factors, the word concreteness effect is also moderated by differences in the surface forms of abstract and concrete words [33], [34]. Specifically, we hypothesize that native English speakers exploit phonological regularities between abstract and concrete words to facilitate lexical access.

### Exploiting Regularities in Word Form: Word Recognition as a Predictive Process

Efficient recognition of spoken words demands that a listener integrates raw acoustic phonetic detail with numerous phonological and semantic expectancies about the incoming data [35]. Much of our understanding about word recognition has been informed by research focusing on aspects of bottom-up processing, although a considerable body of recent emerging work has addressed top-down, expectancy-based contributions. Such top-down expectancy effects are apparent in several domains related to language learning, including learning grammatical distinctions from phonological cues (i.e., syntactic bootstrapping), using transitional phonotactic probabilities to parse word boundaries, and using prosodic and phonetic variation to highlight semantic distinctions during early childhood development [36], [37]–[39]. These cognitive processes–although unique in their own right–are subsumed under the more general rubric of statistical learning, a process wherein listeners exploit regularities at one level of linguistic processing to facilitate higher level linguistic distinctions [40].

Perhaps the most widely accepted form of statistical learning is known as *syntactic bootstrapping* [36], [38], [39]. This cognitive phenomenon operates upon distributional properties of nouns versus verbs across many natural languages. In English, verbs are typically longer and more derivationally complex than nouns; they are less likely to end in a final consonant that is voiced [41]; they are more likely to contain front vowels [42]; and they tend to carry primary stress on non-initial syllables [41], [43], [44]. Syntactic bootstrapping occurs when listeners use the combined strength of many such formal cues to assign a rapid, tentative grammatical parse to the elements of an incoming utterance, a process referred to by Kelly [41] as using sound to solve syntactic problems [42], [45], [46] Monaghan and colleagues [42] showed that computers were able to categorize words and humans were able to categorize nonwords as either verbs or nouns with better than chance accuracy using only phonological (and string frequency) cues.

Another prominent example of statistical learning involves exploiting expectancies about phonotactic probabilities to parse probable word boundaries. Speech perception offers a unique problem for language learning in that speech is typically a continuous signal. Since there are frequently no acoustic “breaks” between spoken words in running speech, one great mystery involves how children learn to efficiently chunk words. Decades of language acquisition research has demonstrated that infants manifest remarkable sensitivity to phonotactic probabilities within their native language and can rapidly induce new regularities within artificial languages [47]–[52]. Saffran and colleagues [48] have argued infants are particularly sensitive to the fact that phonological combinations that occur between words (i.e., transitional probabilities) are highly improbable relative to combinations that occur within words [53], [54]. Transitional probability may therefore act as a relatively stable anchor that allows listeners to chunk words as discrete units embedded within a “sea” of undifferentiated sound [55], [56]–[58].

Theories of statistical learning have garnered mainstream acceptance by satisfying three main constraints. The first prerequisite is that statistical learning must operate on a linguistic regularity that is both statistically informative and discriminative of a higher-level distinction. The second prerequisite for statistical learning is that people must also show sensitivity to the hypothesized underlying regularity. The final prerequisite for is that people must show evidence of moderating effects in various online tasks.

Most approaches to statistical learning have focused on lexical acquisition in childhood. However, it is also apparent that listeners continue to make active use of probabilistic cues throughout the lifespan to aid in many aspects of language processing. For example, Nygaard, and colleagues [59] showed that English monolinguals were faster and more accurate at learning Japanese words when those words were paired with their actual meaning than when they were paired with an incorrect meaning, suggesting that the form of the Japanese word contained cues to its meaning. Farmer, Christiansen, & Monaghan [60] showed that verbs and nouns could be probabilistically differentiated by their surface form alone, and that lexical access times were affected how phonologically typical a word was as a representative of its syntactic class. Our central research question regards ways that healthy adults exploit regularities in the phonological structures of abstract and concrete nouns to facilitate lexical access.

### Arbitrary Symbolism in Lexical Access

Natural language theory often assumes an arbitrary relationship in spoken and written languages between the form of a word and its corresponding meaning [61]. Although arbitrariness is generally a tacit assumption in psycholinguistic research, Wise and colleagues [28] made explicit reference to the phenomenon in a functional neuroimaging study of abstract and concrete word representation, remarking, “Imageability is not apparent in a noun’s phonetic, orthographic, or lexical structure, and so any regional physiological difference results from access to knowledge about concrete and abstract words.”

Arbitrary symbolism makes a clear prediction that word structure will not be informative of word concreteness because phonology and semantics represent two orthogonal domains. Computational models of language processing mirror this assumption by the presence of multiple levels of staged processing (e.g., phonological, lexical, and semantic) [62]–[64]. When hearing a spoken word, for example, we must first process the word’s surface form, followed by word recognition (i.e., lexical access). Listeners can then map meaning onto selected lexical entries and/or near neighbors. This staged process remains serial even in the most interactive models because no mechanism exists for incorporating expectancies about underlying structure directly from word form. Word processing is, therefore, modeled as a series of sequential, independent main effects. We will argue that this assumption of staged processing is not entirely correct with respect to word concreteness and that regularities in word form can potentially allow people to make probabilistic inferences about this particular dimension of meaning (i.e., concreteness) prior to lexical access.

### Foundations for Statistical Learning: Concreteness as a Phonologically Marked Distinction

We first derived distributional evidence for a form-meaning relationship in English from two independent corpus analyses of several thousand nouns [33], [34]. As shown in Tables 1 and 2, abstract and concrete nouns are in many ways phonologically and morphologically distinct. Moreover, these formal cues have strong discriminant power to delineate whether a word represents either an abstract or concrete concept. The prediction of meaning directly from word structure violates a fundamental principle of natural language theory (i.e., arbitrary symbolism) [57], [61]. Linguistic arbitrariness holds that words are only arbitrary symbols for the concepts they denote. As such, arbitrariness makes the explicit prediction there is no reliable relationship between the sound of a word and its corresponding meaning [65].

**Table 1:** Formal Properties of English Abstract and Concrete Nouns.

1	Prefixation is ten times more likely to occur in abstract nouns.
2	Suffixation is four times more likely to occur in abstract nouns.
3	Abstract nouns show higher rates of consonant clustering.
4	Abstract nouns are longer both in total syllables and in phonemes.
5	Compounding (e.g., *bulldog*) is twice as likely to occur in concrete nouns.
6	Concrete nouns are most commonly monomorphemic.
7	Concrete nouns typically hold first syllable stress.
8	Abstract nouns show more variable syllable stress patterns and are more likely to carry non-initial stress as word length increases.
9	Etymologies of concrete and abstract nouns differ significantly. Abstract nouns are most often derived from Latinate. Concrete nouns are more frequently of Germanic origin.
10	Abstract nouns have fewer similar-sounding neighbors (i.e., sparse phonological and orthographic neighborhood density).

**Table 2:** Correlation Matrix of English Noun Psycholinguistic Variables (N = 2,877).

	*NPHN*	*NSYL*	*NMRPH*	*DENS*	*IMAG*	*HFRQ*	*LEX*	*NAME*	*AoA*
NPHN	–	0.89**	0.64**	−0.66**	−0.36**	−0.23**	0.53**	0.55	0.58**
NSYL		–	0.64**	−0.62**	−0.36**	−0.22**	0.52**	0.54	0.58**
NMRPH			–	−0.34**	−0.37**	−0.15**	0.37**	0.38	0.45**
DENS				–	0.25**	0.22**	−0.37**	−0.39	−0.47**
IMAG					–	−0.01	−0.28**	−0.31**	−0.67**
HFRQ						–	−0.61**	−0.47**	−0.40**
LEX							–	0.67**	0.60**
NAME								–	0.60**
AoA									–

*Note*. Pearson correlations represent values for 2,856–2,877 nouns, with the exception of variables correlated with AoA (N = 1477);

** *p*<.001. *FAM* = Familiarity; *NPHN* = Number of phonemes; *NSYL* Number of syllables; *NMRPH* = Number of morphemes; *DENS* = Phonological neighborhood density; *IMAG* = Imageability; *HFRQ* = Hypertext Frequency [106]; *LEX* = Lexical Decision Latency from the English Lexicon Project [66]; *NAME = *Speeded Naming Latency from the English Lexicon Project [66]; AoA = Age of Acquisition value [75].

The two latent factors which appear to drive many of the formal differences that mark abstract and concrete nouns are their language of origin (i.e., etymology) and their derivational complexity. Abstract and concrete nouns emerged in modern English from a different distribution of languages: Concrete nouns tend to be Germanic in origin, whereas abstract nouns were most commonly borrowed from Latinate [33]. These different language families manifest a number of distinct phonotactic patterns and stress placement rules. With respect to morphological complexity, abstract words (e.g., *independence*) tend to be more heavily inflected than concrete words (e.g., *dog*), which tend to be monomorphemic. In addition, English has derived many of its abstract words by inflecting concrete root forms (e.g., *light → enlightenment*). Affixation has phonological consequences in terms of inflating word length and a variety of other acoustic factors, including rate of articulation and phonological neighborhood density. Thus, there exists a tripartite relationship between morphology, phonology, and the semantics of word concreteness (see Table 2 for correlations with word length, morphology, and concreteness). Given that these properties are evident in corpus analyses, in the current study our goal is to ascertain whether listeners actually make use of these statistical relationships during language processing.

We earlier identified two additional constraints for demonstrating the presence of a particular form of statistical learning. Listeners must show both implicit awareness of the interaction and evidence of moderating effects in online tasks (e.g., naming). Our aim in the experiments presented here was to assess both prerequisites in the context of several online and offline language tasks with a focus on the following hypotheses:

- Native English speakers have implicit awareness of a predictive relationship between word form and noun concreteness. Listeners will demonstrate such meta-linguistic awareness by reliably associating acoustic factors such as word length with concreteness in offline tasks (e.g., making judgments of nonwords).
- Listeners exploit regularities in word form to speed lexical access for nouns. When listeners encounter the expected phonological-semantic “match” (e.g., long abstract words, short concrete words), this correspondence will facilitate lexical access. In contrast, when listeners encounter a phonological-semantic mismatch, this irregularity will produce interference in online tasks.

## Methods

We report three experiments and one additional corpus analysis of naming data from the English Lexicon Project [66]. The experiments were conducted over several years across different physical sites (Temple University, University of Florida, and the University of Alberta). The respective institutional review boards at these institutions approved the research, and all experiments were performed with appropriate ethical standards and informed consent. In Experiments 1–3, we examined metalinguistic awareness by manipulating specific acoustic and phonological parameters of nonwords in an offline semantic judgment task. The same participants participated in all three experiments, which were systematically ordered in all possible sequences. In Experiment 4, we examined online performance for real words varied by morphophonological form and concreteness.

## Experiment 1: Judgments of Nonword Concreteness

Participants made yes/no forced choice judgments of word concreteness for aurally presented nonwords varied by a range of phonological and morphological variables. Consistent with our previous corpus analyses and psycholinguistic investigations [23], [34], [67], we hypothesized that listeners would spontaneously associate longer nonword length, higher morphological complexity, and diminished phonological neighborhood density with abstractness. In contrast, listeners would be more likely classify shorter, uninflected nonwords with many similar-sounding neighbors as concrete. The logic of asking participants to make explicit semantic judgments of nonword stimuli is that if there is no underlying association between word form and a particular variable of interest, this task should elicit responses that do not differ from chance performance.

### Participants

Participants included 60 young adults recruited from Temple University (mean age = 24.37 [SD = 9.7] years; mean education = 15.1 [SD = 5.1] years) who were native English speakers and by self-report free of cognitive or language disability.

### Materials

We created a set of 100 pseudowords originally obtained from the ARC Nonword Database [68], and subsequently modified to reflect our manipulation or assessment of the following variables:

- Length in phonemes: Nonwords ranged in length from two (e.g., *ahg*) to 11 phonemes (e.g., *imrúrapentay*).
- Length in syllables: Nonwords ranged in length from 1–4 syllables.
- Syllable stress placement: Half (n = 50) of the nonwords were stressed on the initial syllable; the remainder were stressed on the second syllable.
- Number of consonant clusters: The total number of consonant clusters in each nonword. For example, *zog* has zero clusters, whereas *blorg* has two clusters.
- Phonological neighborhood density: A measure of the number of words that differ from the target word by substitution of a single phoneme. We derived phonological neighborhood density values using the CELEX database [69], by computing the number of first-listed pronunciations with the same stress pattern that were one phoneme difference (by substitution only) from the first-listed pronunciation of the target word.
- Cumulative positional phonotactic probability: This value reflects the sum of the probabilities of each of the individual constituent phonemes of a word appearing in each position relative to all other English words with *any* phoneme in that position. For example, the positional probability of the “sh” in shark is calculated by dividing the number of words beginning with “sh” in first position by the total number of words with any legal phoneme in first position. We derived these values by first translating the pseudowords to a machine-readable analog of the International Phonetic Alphabet known as Klattese. We then submitted all 100 pseudoword entries to the online Phonotactic Probability Calculator at the University of Kansas [70]. Finally, we standardized for word length by dividing the cumulative phonotactic probability by the total number of phonemes in each nonword.
- Cumulative biphone phonotactic probability: Using the same phonetic transcriptions and calculator as described in (e) and (f), we derived the cumulative biphone probability of each nonword identical to the procedure described in (e).
- Affixation: In order to statistically examine the effect of affixation, 36 stimuli were constructed with legal English prefixes (e.g., *inhighosht*).
- Wordlikeness: One possibility is that participants classified nonwords based solely on their phonological plausibility or representativeness as a legitimate form within the English lexicon. This phonological familiarity phenomenon in nonword processing has been formally described as *wordlikeness* [71]. In a posthoc analysis, we collected ratings of wordlikeness from 25 native-English speaking adults (mean age = 25, 8 males) by adapting the Frisch et al. [71] Likert rating scale. Participants read the following instruction, “Please rate the extent to which each item below represents a plausible English word. You will scale your judgments from 1 (Low-Impossible: This word could never be a word of English) to 7 (High-possible)–this word could easily be a word of English. Please work as quickly as possible without sacrificing accuracy.”

A female native speaker of American English read aloud the 100 nonwords from their broad transcription in the International Phonetic Alphabet (IPA). We digitally recorded her production. Transcriptions and audio WAV files are freely available by contacting the authors.

### Procedure

Participants were tested in a quiet room with no direct view of the examiner. After providing informed consent, participants were informed that they would hear unfamiliar words constructed from a variety of foreign languages. Their task was to signal yes/no via mouse click to the written instruction: “Can you see, hear, smell, touch, or feel this?”.

Participants were seated at a Windows-based PC computer and fitted with stereo headphones. We standardized stimulus delivery and recorded response latencies and accuracies using E-Prime 1.0 software (Psychology Software Tools, Inc., Sharpsburg, PA). After completing a brief familiarization sequence, participants heard the 100 nonwords presented in fully randomized order. After each nonword, participants signaled their yes/no response via mouse click. The stimuli advanced with a 1000 ms delay. We imposed no time restriction for the task.

### Statistical Design and Data Analyses

We conducted a stepwise multiple linear regression in order to examine the weighted contribution of phonological and morphological predictors toward judgments of nonword concreteness. The dependent measure was concreteness agreement for each nonword as measured by the total number of participants (of 60) that classified each nonword as concrete. Agreement that differed from chance (p<0.05) was interpreted to reflect systematic use of word form information. For example, 49 participants judged *strope* as concrete (binomial p = .006) whereas only 15 participants judged *ipskvingem* as concrete (binomial p = .001). Item level agreement provides an index of the representative strength of each stimulus as a phonological exemplar of the semantic distinction of concreteness.

We assessed potential multicollinearity violations using SPSS 18′s Variance Inflation Factor (VIF) algorithm; a VIF>10 necessitates de-correlation procedures. At each step during the regression here, the VIF was <5. In light of this diagnostic, we did not pursue data transformation or factor reduction.

We also conducted a polynomial trend analysis examining the nature of the relationship between word length and concreteness. We predicted that participants would demonstrate a strong relationship between length and their perception of abstractness. Since our earlier pilot work showed evidence of a non-linear association between these variables [72], we conducted a polynomial trend analysis in an attempt to assess the best fit of both linear and higher order (e.g., quadratic, cubic) relationships.

### Results

Table 3 summarizes the results of the regression. The second step yielded two significant predictors: total number of syllables and wordlikeness. Variables excluded from the model were stress placement, pseudomorphology, number of consonant clusters, phonological neighborhood density, phonotactic probability, and biphone phonotactic probability. Together, total syllables and wordlikeness accounted for 67% of the variance in the concreteness judgments of nonwords [F(3,99) = 93.10, p<.001].

**Table 3:** Stepwise multiple regression for variables predicting nonword concreteness.

	Step 1			Step 2		
Variable	*Beta*	*SE*	*â_stan_*	*Beta*	*SE*	*â_stan_*
N-Syllables	−7.46	.62	−.77	−4.46	.91	−.46
Wordlikeness				2.59	.61	.40
*R^2^*	.59			.66		
*F-Value & Df*	143.17 (2,99)			93.10 (2,99)		
*P-Value*	<.001			<.001		

*Note: Beta* reflects the unstandardized beta coefficient; *â_stan_* reflects the standardized beta coefficient. Predictors included in Step 1: N-Syllables; Step 2: N-Syllables, Wordlikeness; Variables excluded from the final model: phonological neighborhood density; morphology; single segment phonotactic probability; cumulative biphone phonotactic probability; N-consonant clusters; syllable stress placement.

**Figure 1:**
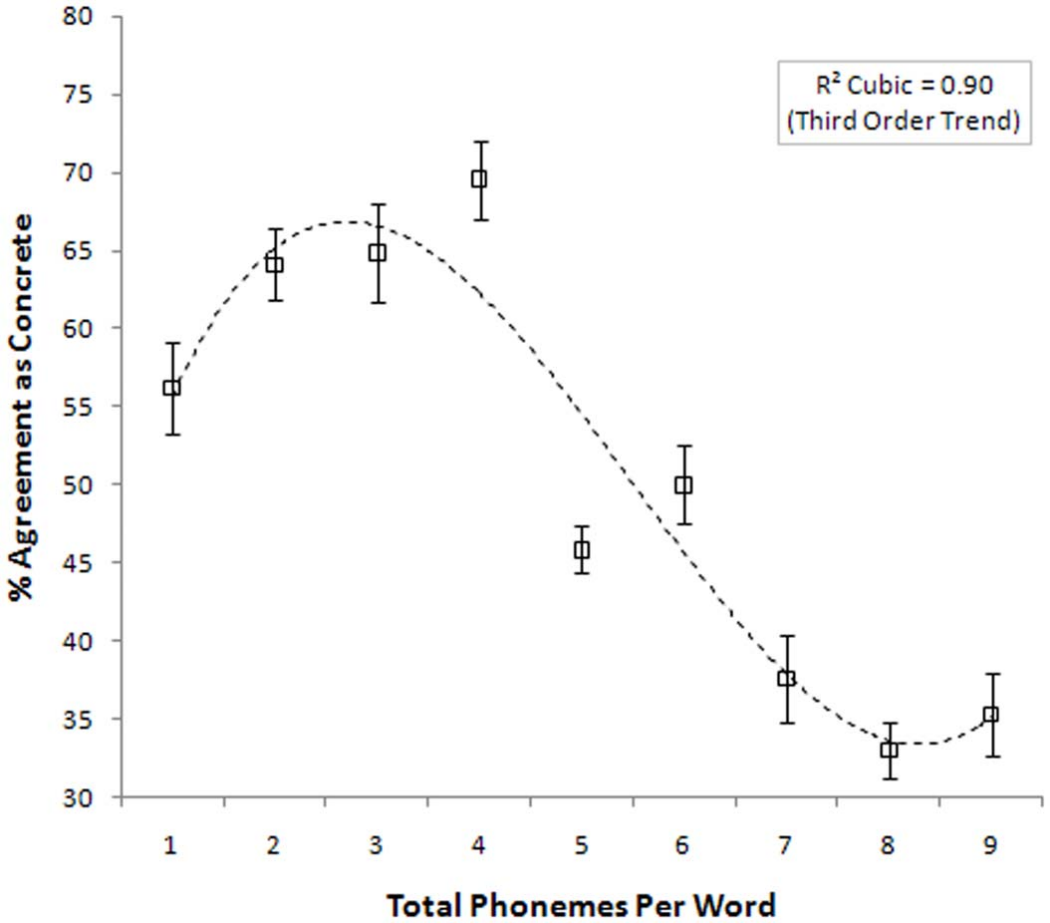
Nonword Concreteness Agreement.

Figure 1 illustrates the trend toward abstractness as nonwords increased in length and phonological complexity while simultaneously decreasing in phonological neighborhood density. As the reader will note, this relationship was markedly nonlinear as confirmed by the polynomial trend analysis which best fit a cubic function to the data [cubic trend r = .90], reflecting the fact that participants did not classify the shortest nonwords as concrete.

### Interim Discussion: Experiment 1

We set out to test the hypothesis that listeners are sensitive to formal cues that mark abstract and concrete words. Participants confirmed this assumption by demonstrating agreement at levels far beyond chance in assigning meaning to nonwords. In general, listeners showed a strong tendency to associate longer nonwords with abstract concepts. However, this length-concreteness relationship was nonlinear in that many of the shortest nonwords (e.g., two phonemes long) were often rated as abstract. One might speculate that this nonlinearity reflects the influence of English function words such as *the*, *of*, and *a.* Function words are invariably short and tend to be ubiquitous in terms of lexical frequency. In addition, function words are also regarded as highly abstract in that they do not invoke strong sensory imagery. It is therefore plausible that participants manifest sensitivity to this distributional property imposed by English function words.

Multiple regression isolated unique variance due to word length, but this statistical procedure also afforded insight into alternate strategies. For example, it makes it possible to assess whether it is likely that phonological familiarity could be driving performance in such a way that participants could circumvent the task altogether by classifying any phonologically implausible item as “abstract”. Comparison of the partial correlations and beta weights for word length and wordlikeness suggests that this was not the case. However, this conclusion must be interpreted with caution. The nature of the English phonological system is such that many of the predictors we entered into the model, although not technically in violation of the multicollinearity, are irreducibly correlated (e.g., as word length increases, phonological neighborhood and wordlikeness also diminish) [73]. Nevertheless, this experiment does provide evidence that no single factor such as morphology (i.e., affixation), phonological neighborhood density, or wordlikeness exclusively accounts for agreement.

## Experiment 2: Effects of Acoustic Duration and Syllable Length

Our aim in this experiment was to further decompose the effects of word length on associations with the concreteness of nonwords. We examined two aspects of word length: acoustic duration and total number of syllables. Our rationale for manipulating these factors independently is that the correlation between the number of syllable constituents in a word and that word’s acoustic duration is imperfect. For example, *ping* and *pong* are both monosyllabic words, but their differing vowel durations cause them to acoustically unfold over different periods of time. There exists compelling evidence to suggest that these two word length variables (i.e., duration and total number of syllable constituents) exert independent effects [74].

We employed nonword stimuli to circumvent the potentially contaminating effects of word meaning. We additionally noise-distorted nonwords by applying a low-pass filter (cutoff 800 Hz) with the goal of stripping much of their phonological detail. This frequency threshold was chosen to ensure that listeners could discern the number of constituent syllables and other duration cues but could not reliably perceive phonemic information. This noise-distortion procedure produced stimuli perceptually analogous to speech heard while underwater. Noise distortion allowed us to isolate two variables of interest with respect to word length: acoustic duration and number of syllables by systematically controlling many other psycholinguistic variables (e.g., phonological neighborhood density, wordlikeness).

### Methods

#### Participants

Participants included undergraduates (N = 50; 37 female) recruited from two sites, the University of Alberta (n = 40) and the University of Florida (n = 10). Mean age was 20.2 years (SD = 3.4), with an average of 13.7 (SD = 4.2) years of education. By self-report, participants had no history of language learning disability and were native English speakers. Participants provided written informed consent in accord with the institutional review boards of the University of Alberta and the University of Florida.

#### Materials

We factorially varied nonwords by syllable length (1, 2, 3, or 4 syllables) and vowel duration (short/long). This manipulation yielded 20 nonwords at each syllable length (80 total nonwords). Half the stimuli were composed of short vowels (e.g.,/I/as in pit) and voiceless consonants. The remaining stimuli were composed of long vowels (e.g.,/o/as in goal) and voiced consonants. We maintained syllabic stress on the initial syllable and also matched on the total number of phonemes at each syllable length. We also matched volume across stimuli using the GoldWave acoustic waveform editor’s root mean square (RMS) amplitude matching function. Stimuli were digitally recorded with a 44.1 kHz sampling frequency and then distorted by applying a lowpass filter with a cutoff of 800 Hz (steepness of +10). This low pass filtering procedure reduced speech intelligibility such that consonants were fully unintelligible. Filtering did, however, preserve some acoustic and phonetic features such as aspiration, amplitude, and pitch alternation that provided intermittent cues to vowel identity. For the filtered set, participants could discern syllable stress placement and total number of syllables but few if any individual consonants or phonotactic combinations.

Participants in both locations heard stimuli through over-the-ear headphones. At the University of Florida we used E-Prime 2.0 for stimulus delivery and to collect response times. At the University of Alberta we used ACTUATE software (Westbury, 2007) for stimulus delivery and to collect response times.

#### Procedures

Listeners were seated in a quiet laboratory setting at a computer with the experimenter out of direct view. Participants were told that they would soon hear scrambled words and that their task was to answer yes/no to the question: “Is this something you can see, hear, taste, touch, or smell?” as quickly as possible. Following a brief familiarization composed of four practice trials, participants heard each of the 80 stimuli in completely randomized order. On each trial, participants heard the garbled stimulus at the end of a short carrier phrase announcing, “The next word is…” Listeners signaled their yes/no judgment by pressing “yes” or “no” keys on the keyboard, and we counterbalanced the response key placement (right/left). We imposed no time restriction for this task, and the inter-trial interval was 1500 ms.

#### Statistical design and data analyses

We contrasted response latencies and accuracies using two separate 2-factor nested repeated measures ANOVA by subjects (F_1_) and by items (F_2_). The first factor was nonword length in syllables (short or long) nested within the acoustic duration factor (short or long). We eliminated reaction time outliers corresponding beyond two standard deviations from the mean of each participants RT distribution. We also eliminated reaction times faster than 500 ms. After data trimming, the analyses retained >98% of the original items.

### Results

**Figure 2:**
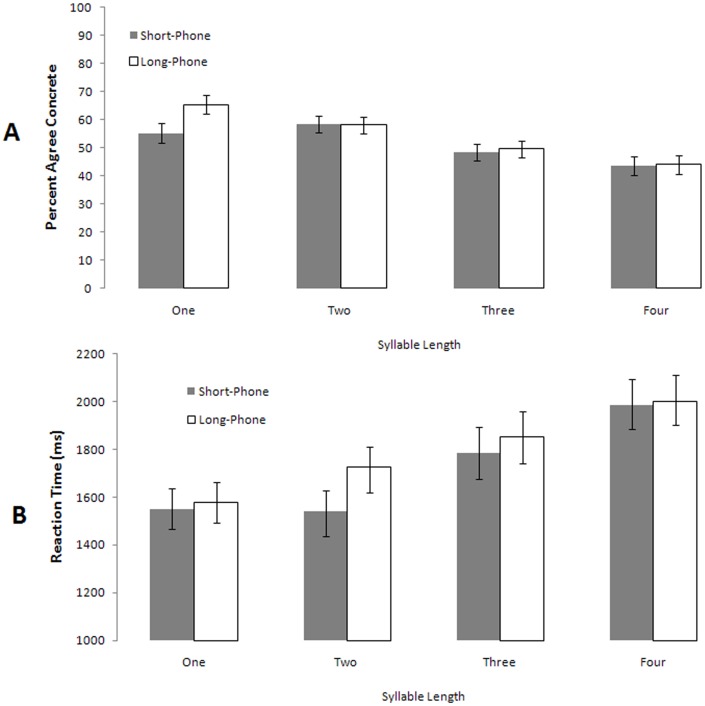
Nonword agreement as functions of acoustic duration and syllable length.

Reaction time and nonword concreteness agreement results appear in Figure 2.

Participants showed a trend toward an interaction between syllable length and phoneme duration by subjects [F_1_(3,147) = 2.5, p = .06] and a significant interaction between these variables at the item level [F_2_(3,72) = 2.74, p = .05]. Main effects analyses revealed that listeners strongly associated increasing syllable length with abstractness but showed no sensitivity to the manipulation of acoustic duration as evident in large main effects of syllable length [by subjects F_1_(3,147) = 10.44, p<.001, ç^2^ = .18; by items F_2_(3,79) = 27.64, p<.001, ç^2^ = .54]. Judgments of concreteness were characterized by a significant negative linear trend across word lengths [by subjects F_1_(1,49) = 1.73, p<.001, ç^2^ = .24; by items F_2_(3,79) = 71.26, p<.001]. The more syllable constituents a word had, the more likely it was to be classified as abstract. In contrast, the main effect of acoustic duration approached but did not reach significance [F_1_(1,147) = 2.96, p = .09; by items F_2_(3,79) = 3.83, p = .054]. Thus, participants tended to rate stimuli differing by duration but matched on total number of constituents (e.g., *ping* and *pong)* with roughly comparable agreement.

With respect to reaction times, there was a significant interaction between syllable length and phoneme duration by subjects but not by items [F_1_(3,147) = 3.08, p = .02; F_2_(3,82) = 1.88, p = .13]. Participants were sensitive to a significant main effect of syllable length, with reaction times increasing as a linear function of syllable length [F_1_(1,49) = 160.85, p<.001, linear term F_1_(1,49) = 160.81,p<.001, partial ç^2^ = .76; by items F_2_(1,78) = 69.71, p<.001]. Reaction times were also characterized by a main effect of acoustic duration. Participants identified acoustically shorter items faster than longer items by an average margin of 64 ms [F_1_(1,147) = 14.54,p<.001, partial ç^2^ = .30]; however, this difference was not significant by items [F_2_(1,79) = 2.0, p = .16].

### Interim Discussion: Experiment 2

In this experiment we stripped much of the acoustic-phonetic information that was available to listeners. This allowed us to isolate two potentially dissociable factors related to word length: total number of syllable constituents and acoustic duration. Participants demonstrated sensitivity to syllable length but failed to show robust sensitivity to acoustic duration. Participants behaved more systematically at shorter lengths (e.g., one syllable nonwords) with agreement that approached chance at longer durations. These data do, however, reflect considerable inter-subject variability. The task was difficult in that it required participants to make judgments of meaning for what many perceived to be random fragments of noise. A number of participants signaled randomly. We did not eliminate these responses because they were in fact meaningful; this subset of participants failed to discern the hypothesized pattern. Thus, the observed effect sizes are small, but the observation of a systematic negative linear trend (i.e., more syllables = more abstract) does implicate a pattern beyond chance responding. This response pattern is also consistent with our previous distributional findings showing that abstract words tend to in fact be longer than concrete words in total syllable constituents [33], [34].

## Experiment 3: Derivational Complexity

In this experiment, we examined the effects of affixation on accuracy of real word semantic judgments of concreteness. We specifically assessed bias that participants manifest toward processing morphologically complex words as abstract, predicting greater error rates for semantic-morphological mismatches such as inflected concrete nouns (e.g., *professor*) and uninflected abstract nouns (e.g., *liberty*) relative to the canonical pattern of affixation revealed through corpus analyses (i.e., inflected words tend to denote abstract concepts).

### Methods

#### Participants

Experiment 3 involved the same participant pool as described in Experiment 2.^FN1^.

#### Materials

We factorially crossed noun concreteness by morphological complexity, resulting in the following cells (20 words per cell): a) concrete uninflected (e.g., *hurricane*); b) concrete inflected (e.g., *professor*); c) abstract uninflected (e.g., *liberty*); and d) abstract inflected (e.g., *conclusion*). Stimuli within each of these conditions included nouns (N = 80) with concreteness ratings from the MRC Psycholinguistic database [75]. Half of the nouns were abstract, half were concrete. Abstract nouns had a mean concreteness rating of 293 on a 100−700 scale; concrete nouns had a mean concreteness value of 550 [abstract-concrete contrast: t(77) = 21.75, p<.001]. Within each of the concreteness conditions, half of the stimuli were polymorphemic (i.e., each word had at least one prefix or suffix in addition to the root). The remainder was monomorphemic, consisting of root forms only. Among abstract nouns, the morphologically inflected and uninflected stimuli did not differ by their rated concreteness values [mean uninflected = 293, inflected = 294 (on 700 point scale), t(37) = .06, p = .95]. Analogously, concrete nouns that differed by morphological complexity were matched on rated concreteness [mean uninflected = 566, inflected = 534, t(38) = 1.78, p = .08].

Stimuli were matched on length at 3 syllables. Stimuli were also assessed or matched on the following additional psycholinguistic variables:

- Word frequency: Conditions did not differ by lexical frequency as assessed by the Gent SUBTLEX psycholinguistic database norms [mean frequency = 11.65 per-million words; matching statistic F(3,79) = .61, p>.05] [76].
- Familiarity: We assessed familiarity in a posthoc analysis by via ratings from the MRC Psycholinguistic database [75]. The grand mean for familiarity across the four experimental conditions was 489 (on a 100−700 point scale). Familiarity was slightly higher for one condition [abstract inflected mean = 524 on a 700 point scale] relative to matched values on the other conditions [F(3,79) = 3.65, p = .01]. This difference in familiarity, although statistically significant, represents a very small effect (z = 0.36) when considering the range of familiarity for all words in the MRC database [mean familiarity = 488, s.d. = 99].
- Phonological uniqueness point: We calculated the phonological uniqueness point using the CELEX database [69]. For each word, we found the point in the accent-marked phonological representation at which only the word (and its inflections) was a possible completion. We then located the point corresponding to the end of that phonological stem in the word’s sound file (manually), and used its distance from the start of the file (in ms) as a measure of the word’s phonological uniqueness point.
- Semantic/Lexical neighborhood size: We assessed semantic neighborhood size across conditions in a post hoc analysis by computing the Average Radius of Co-occurrence (ARC) using the HiDEx model of word co-occurrence [77], [78]. A word’s ARC is a measure of co-occurrence neighborhood density, defined as the average similarity in co-occurrence space of all the co-occurrence neighbors that fall within a pre-defined radius around that word (or the distance to the nearest neighbor, if no words fall within the pre-defined radius). Because HiDEx allows for alterations to its parameter settings the radius size used for computing ARC must be determined dynamically based on systematically sampling billions of random word pairs for any particular parameter set. Although conditions did in fact differ by ARC density, this variable was uncorrelated with judgment accuracy [Pearson r = −.10, p>.05], suggesting that these baseline differences cannot alone account for the observed pattern of results.

We recorded stimuli as individual WAV files.

#### Procedure

Participants were seated in a quiet laboratory setting and fitted with headphones. Once we ensured a comfortable listening volume, the experimenter was positioned out of view. Participants read self-paced instructions directing them to signal yes/no by pressing corresponding buttons to the question, “Is this something you can see, hear, smell, touch, or feel?” We counterbalanced the response key placement (right/left), and participants were instructed to respond as quickly and accurately as possible upon hearing a word. After completing a series of four familiarization trials with feedback on accuracy and response time, participants made concreteness judgments for the 80 stimulus items presented in completely randomized order. We imposed no time restriction for the semantic decision; there was a 1500 ms inter-trial interval.

#### Data analytic procedures and statistical model

We contrasted response accuracies for semantic decisions using two separate 2-factor repeated measures ANOVAs by subjects (F_1_) and by items (F_2_).

### Results

**Figure 3:**
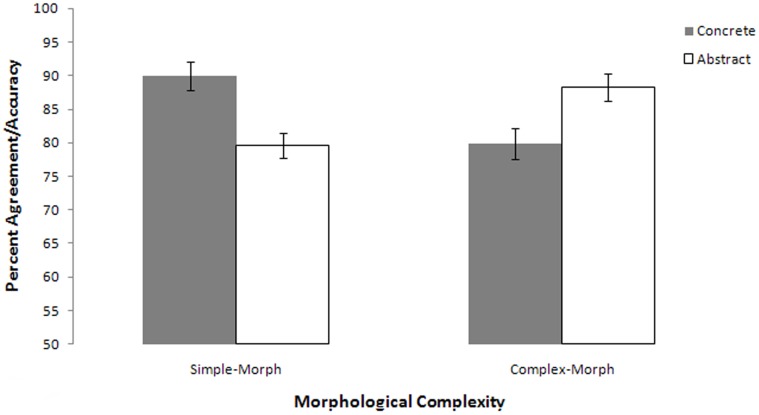
Single word semantic judgment accuracy and reaction time as functions of word length and concreteness.

Figure 3 summarizes accuracy results. Participants demonstrated a crossover interaction between the concreteness of the target word and its morphological complexity [F_1_(1,49) = 93.84, p<.001, ç^2^ = .13; by items F_2_(1,76) = 7.91, p = .01, ç^2^ = .09]. Among concrete nouns, participants were more likely to misclassify a morphologically complex word (e.g., *professor*) as abstract [t_1_(49) = 6.73, p<.001; by items t_2_(38) = 2.07, p = .04]. In contrast, when encountering an abstract uninflected word (e.g., *liberty*), participants were more likely to misclassify the target as concrete [t_1_(49) = 6.25, p<.001, trend by items t_2_(38) = 1.91, p = .06].

### Interim Discussion: Experiment 3

In this semantic judgment task, participants showed evidence of using form to aid in judgments of meaning, tending to mis-classify non-canonical words (i.e., words whose form and concreteness are mismatched). This error is logical from the standpoint of distributional probability in that a derivationally complex word such as *professor* should denote an abstract concept. However, words such as *professor* represent a form-meaning mismatch in that most people tend to rate *professor* as highly concrete [75], [79].

## Experiment 4: Concreteness × Phonology Interaction Effects in Word Naming

The experiments we have reported thus far have involved offline judgments of nonwords or online judgments of a small and carefully selected real word item pool. These tasks are valuable from the standpoint of experimental control, but they also lack ecological validity: It is highly unlikely that a person might find himself in a situation where he is called upon to make semantic judgments of acoustically-distorted nonwords. Therefore, in the final experiment to follow we assessed the presence of a form × concreteness interaction within the context of the more naturalistic online task of word naming. Specifically, we investigated whether the magnitude of the concreteness effect in naming is moderated by word form.

Naming latency is among the most extensively investigated psycholinguistic domains related to the word concreteness effect. Most studies have reported a small but consistent reaction time advantage for naming concrete relative to abstract words [10], [13], . However, other studies have shown that this concreteness advantage is either eliminated or reversed under certain circumstances related to subject-level factors (e.g., poor reading ability) or item-level differences (e.g., spelling-sound consistency, age-of-acquisition effects, sentence context) [23], [34], .

Controversy persists as to the nature of the concreteness effect in naming because the magnitude of the effect is in fact so variable. The vast majority of naming studies to date have employed restricted item pools carefully matched on psycholinguistic variables (see Experiment 3 here). The English Lexicon Project [66] is a psycholinguistic database that can potentially ameliorate such small sample bias inherent in this approach through analyses of trial level naming data for many thousands of words [75], . In the experiment to follow, we analyzed naming reaction times as the dependent variable in a large multiple regression. Predictors included a variety of phonological and semantic variables in addition to a series of form × concreteness interaction terms. The data in this regression included thousands of English nouns with imageability ratings.

### Methods

We first isolated a large corpus of English nouns (N = 2, 852) and after eliminating archaic, obscure, and syntactically ambiguous entries, we coded each noun on the following psycholinguistic variables:

- Length in phonemes – We counted total phonemes-per-word
- Length in syllables – We coded total number of syllables-per-word.
- Word frequency – Log transformed values from the Lund & Burgess (1996) hypertext frequency database [66].
- Phonological complexity *-* We coded a coarse measure of phonetic complexity by counting the total number of consonant clusters per word.
- Phonological Neighborhood Density – We obtained density values from the Washington University Neighborhood Density Database [93].
- Cumulative Phonotactic Probability – for method see experiment 1
- Cumulative Biphone Phonotactic Probability – for method see experiment 1
- Morphological Complexity – We counted raw totals of stems and affixes for each word. For example, *disagreement* can be decomposed into a stem *(agree*), prefix *(dis-*), and suffix (*-ment*), for a total of three morphemes.
- Concreteness *–* We queried word concreteness values from the MRC Psycholinguistic database [75].
- Familiarity *–* We queried familiarity values from the MRC Psycholinguistic database.
- Speeded Naming Latency – We queried trial level naming latency data for each word from the English Lexicon Project (hereafter ELP) database [66]. Naming latencies reflect the interval between presentation of a written word and onset of articulation for naming the word aloud as captured through a microphone relay.
- Onset Phonetic Features – A significant proportion of the variance of speeded naming is derived from phonetic features of word onsets such as consonant voicing that influence sensitivity of the voice key microphone relay. Prior to interpreting a variable of interest (e.g., word frequency), a common methodological practice involves partialling out the variance introduced by the initial phoneme of each word through hierarchical regression [94], [95]. We did so here by using the phonetic feature coding system developed by Balota & Yap [90], [91]. This system consists of a dichotomous phonetic feature matrix where each target word is coded for the presence or absence of the following variables characterizing the voice, place, or manner of articulation: ± voice, ± bilabial, ± labiodental, ± dental, ± alveolar, ± palatal, ± velar, ± glottal, ± stop, ± fricative, ± affricate, ± nasal, ± liquid/glide.

#### Principal components analysis/factor reduction procedure

Our hypothesis is that the cumulative strength of a range of formal factors interacts with the semantic properties of a word in the service of lexical access. One might empirically test this hypothesis by modeling interaction terms between formal variables and word concreteness. Regression is an optimal framework for this endeavor because it honors the continuous nature of the data (e.g., concreteness is a graded phenomenon). However, unlike a factorial univariate approach such as ANOVA, linear regression requires the user to specify a set of interaction terms. The choice of how to model such interaction terms is not trivial. One might, for example, choose to model all possible 2-way interaction terms by simple multiplication of concreteness with each phonological or morphological variable (e.g., concreteness × number of phonemes, concreteness × number of morphemes, etc.). However, this approach is costly in terms of data proliferation and potential multicollinearity problems. For those reason, we chose to first pursue factor analysis with the goal of reducing and de-correlating the original set of predictors. We did so by submitting the following formal variables to a principal components analysis (PCA) with Varimax rotation: familiarity, frequency, phonological complexity, number of phonemes, number of syllables, phonological neighborhood density, cumulative phonotactic probability, cumulative biphone phonotactic probability, syllable stress placement, and morphemes per word.

The PCA extracted four principal components (see Table 4), corresponding to a linear combination of what might roughly be considered 1) word length (subsuming total syllables, phonemes, morphemes, and stress); 2) phonological probability (subsuming phonotactic probability and biphone probability); 3) lexical-semantic availability (subsuming frequency and familiarity); and 4) phonological complexity (subsuming density and rates of consonant clustering). These four components explained 81% of the variance in the original data.

**Table 4:** Factor analysis/Component matrix for phonological and morphological variables.

Predictor	Component			
	Factor 1	Factor 2	Factor 3	Factor 4
N-Syllables	**.92**	.04	−.16	−.01
N-Phonemes	**.89**	.04	−.14	.28
Syllabic Stress	**.77**	.00	.04	−.12
N-Morphemes	**.78**	.05	−.05	.00
Biphone Phonotactic Probability	−.01	**.99**	−.02	.02
Phonotactic Probability	.13	**.98**	−.05	.05
Word Frequency	−.12	.06	**.90**	−.02
Word Familiarity	−.10	−.12	**.90**	−.02
Phonological Neighborhood Density	−.61	−.13	.21	**.42**
Phonological Complexity/Clustering	.03	.03	.01	**.95**

Note: The above component matrix was derived using SPSS-18′s factor analysis algorithm employing a Varimax rotation with Kaiser normalization. The rotation converged after four iterations.

Based on these four reduced factors, we created a subset of phonology × concreteness interaction terms by first adding a constant to all values to ensure that the factor loadings were made positive. We then multiplied the value of each word’s factor score by its respective concreteness rating. This procedure yielded three new regressors representing the following multiplicative interaction terms: a) length × concreteness; b) phonological probability × concreteness; and c) phonological complexity × concreteness.

#### Multiple regression procedure

We conducted a hierarchical multiple regression predicting naming latency for 2877 English nouns. In step one, we entered the 13 dichotomous onset variables (e.g., ± voicing, ± fricative, etc.) as a means for removing the variance of this factor. In step 2, we entered 8 predictors of interest, including: Word Concreteness; Word Length (Factor 1); Phonological Probability (Factor 2); Lexical Availability (Factor 3); Phonological Complexity (Factor 4), and the three interaction terms created in the previous step.

### Results

**Figure 4:**
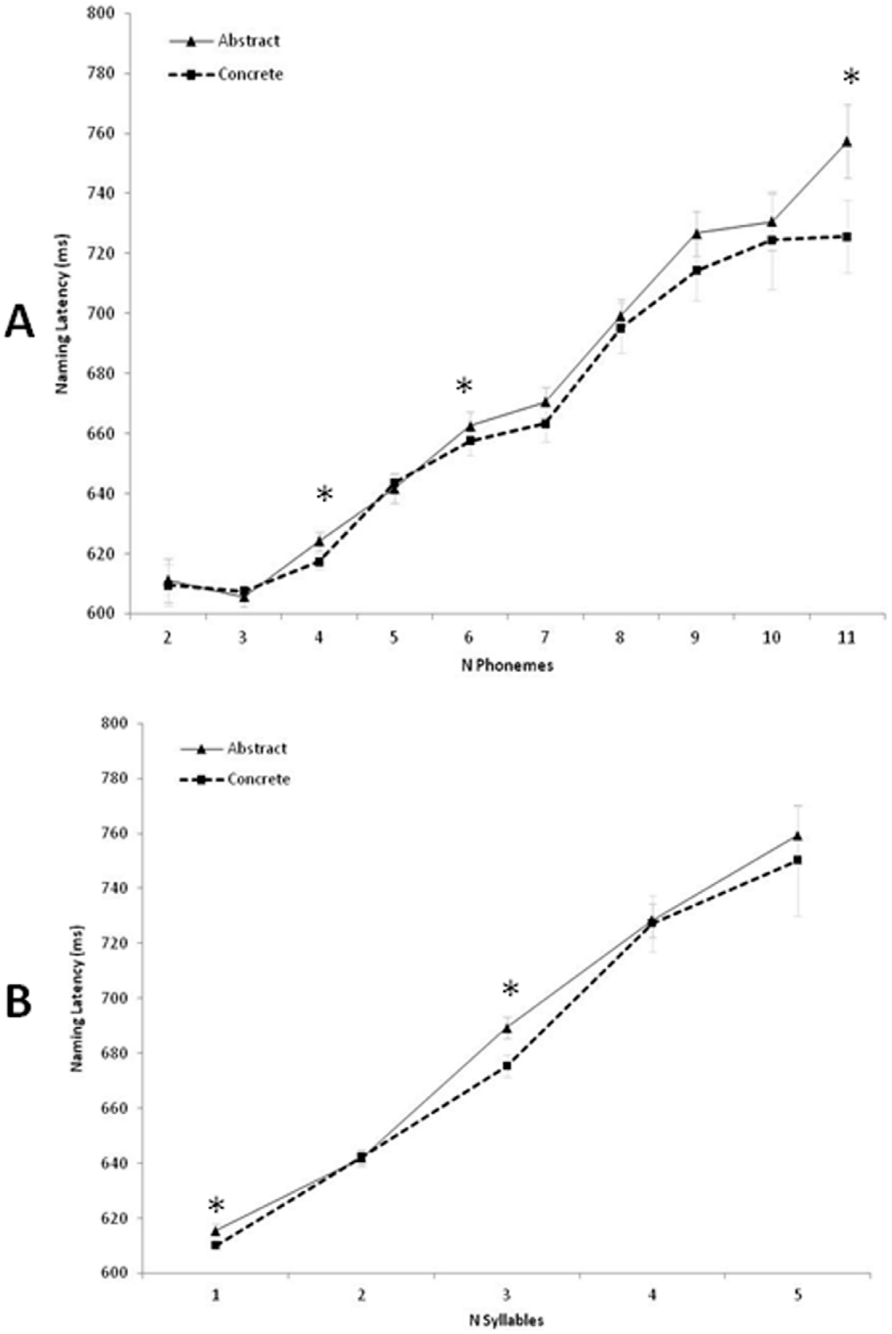
Concreteness *form interaction effects in English noun naming. Note: The graphs represent naming reaction times as functions of word concreteness. For visual presentation we binned abstract and concrete words via a median split on word concreteness: abstract <492 (on a 700 point scale) < concrete. Panel A represents reaction time differences for abstract versus concrete nouns matched across different phoneme lengths. Panel B represents reaction time differences for abstract versus concrete nouns matched across different syllable lengths.

The initial step of the model (onsets only) accounted for 10% of the variance of the data (R^2^ = .099); inclusion of the remaining variables of interest in step 2 accounted for 54% of the variance (R^2^ = .539). Table 5 summarizes the results of the regression analysis, and Figure 4 depicts the relation between specific phonological variables and concreteness. Each of the four factors was predictive of speeded naming latency, as were the interaction terms for phonological complexity × concreteness and phonotactic probability × concreteness.

**Table 5:** Hierarchical multiple regression table predicting speeded naming latencies.

Variable	Beta	SE B	â	t-value	p-value
Factor 1: Word length	34.27	3.02	.49	11.35	<.001
Factor 2: Phonotactic probability	−11.92	3.55	−.71	−3.35	.001
Factor 3: Frequency/Familiarity	−33.02	.92	−.47	−35.75	<.001
Factor 4: Phonological complexity	19.91	3.10	.29	6.43	<.001
Length Factor *Concreteness	−.002	.01	−.01	−.21	.831 (ns)
Probability Factor *Concreteness	.03	.01	.29	4.17	<.001
Complexity Factor *Concreteness	−.04	.01	−.38	−5.57	<.001

Note: Values above reflect significance for step 2 of the regression model after partialling the variance due to word onsets (see description of step 1); Final model R = .74, R2 = .54, Model significance F(18,2757) = 178.27, p<.001.

When comparing the mean naming reaction times for nouns grouped by a median split on their concreteness ratings, there is a statistically significant concreteness effect on the order of a 30 ms speed advantage for initiating naming of a concrete word [F(1,2769) = 103.81, p<.001, partial ç^2^ = .04]. The mean reaction time for naming a concrete noun is 634 ms [s.d. = 60 ms], whereas the average latency to name an abstract noun is 660 ms [s.d. = 77 ms]. Nevertheless, these grand mean comparisons obscure relations between word form and concreteness. As evident in Figure 4, the magnitude of the concreteness effect is highly variable across different dimensions of word form. It was not uncommon to encounter virtually no abstract-concrete reaction time difference and in some cases, participants paradoxically showed a marginally faster naming latency for abstract words.

### Experiment 4: Interim Discussion

The ELP can elucidate word concreteness effects on a scale never before possible. The most common finding to date is that concrete words are faster and more accurately named than their abstract counterparts, although a sizeable proportion of previous studies have failed to detect this concreteness effect. Our corpus analyses offer a potential latent factor underlying such discrepancies in that the magnitude of the concreteness effect in naming is moderated by word form. One might contextualize the theoretical relevance of this moderating effect in terms of the very definition of an interaction. The effect of concreteness depends on a word’s form. Conversely, the interpretation of word form effects in the naming task must also account for meaning.

This simple statement has far reaching implications for both theory and method in psycholinguistic research. Consider, for example, a hypothetical study where one arbitrarily chooses to match stimuli at two syllables with the goal of contrasting semantic differences between a pool of concrete and abstract words. Figure 4 illustrates a pitfall with respect to this common experimental control. The choice of matching items at two syllables will likely yield virtually no concreteness effect given a sufficiently large item pool. In contrast, the decision to match at three syllables will potentially exaggerate the concreteness effect.

We hypothesize that people use formal factors such as word length and morphological complexity to speed the course of lexical access for abstract and concrete words. In previous work we found that these effects are most apparent in the auditory modality (e.g., auditory lexical decision, auditory rhyme judgments, auditory semantic decisions) [23], [34], [72]. The ELP naming latency results here indicate that interaction effects may also extend to visual word recognition. However, these effects are weaker for written words. The strongest test of our hypothesis is that naming RTs would show a crossover interaction similar to that seen in Experiment 3, in which concrete words were identified faster when they were short and derivationally simple, whereas abstract words more rapidly named when they were long and derivationally complex. Participants failed to produce a clean crossover interaction but instead showed variable attenuation and exaggeration of the concreteness reaction time advantage across different phonological dimensions.

There are numerous alternative explanations for this finding. One possibility is that our indices of formal complexity (e.g., word length, total morphemes) are too coarse and that readers use an entirely different set of probabilistic cues than listeners (e.g., visual word form complexity). A related possibility is that the hypothesized effects are isolated exclusively to auditory word recognition. Finally, it is possible that the hypothesized effects are idiosyncratic to a particular grammatical class (e.g., verbs) or emerge only in real world online sentence contexts. These all remain open empirical questions.

## Discussion

Statistical learning is a phenomenon that links these disparate levels of linguistic processing by allowing listeners to make inferences about one domain based on input from an entirely different modality (e.g., inferring syntax from phonology). The links and features required for such inferencing are rarely explicit in models of spoken and written language comprehension. Neither dual route models [96], nor parallel distributed models [97], [98] explicitly implement many features to which readers are known to be sensitive, such as morphological complexity, morphological family size, semantic or orthographic/phonological neighborhood size, stress patterns, or word length. In models that emphasize a concrete lexical-level representation, such as dual route models, such features could be implemented somewhere within (or between) the letter input stage and the stage of accessing the orthographic input lexicon. However, they would sit uneasily with the simplicity of such models, essentially requiring a radical transformation of those models by positing a series of connections between subword elements and semantic access that are not currently part of the model. Parallel distributed models can more easily account for features that depend upon computable formal properties of letter strings. In such models, it is already assumed that there is a high degree of interactivity between orthographic and phonological subword elements during the process of lexical access. The influence on semantic access of patterned co-occurrence information from these computations is unproblematic, since such models assume an interactivity between phonology, orthography and semantics and often implement sub-word level features. More recent dominant models of lexical access and language acquisition are also premised upon a high degree of interactivity between phonological, semantic, and syntactic levels of processing [99], [100].

Probabilistic inference from patterns in subword elements appears to be highly adaptive for early word learning as well as for lexical processing in the mature language user. Established approaches to statistical language learning have built support by satisfying several overarching criteria. The most basic criteria are that a reliable pattern must be present in the data, and that pattern must impact language processing. In our earlier corpus studies we demonstrated that patterns do indeed exist within the data: formal structures of words are indeed predictive of word concreteness. Recently, Piantodosi and colleagues provided further support for this claim through one of the most computationally extensive corpus analyses to date. The authors queried more than a trillion words across unrelated languages [101]. Based on distributional frequency, the authors argued that natural languages optimize word length as a function of information content such that longer words convey more information content than shorter words [102]. In a reply to the authors’ original study, we posed the question of whether this relationship between word length and information content could then logically extend to grammatical class and word concreteness [103]. Across many languages verbs tend to be longer than nouns, and abstract nouns tend to be longer than concrete nouns. If word length is optimized for information content, does this somehow imply that verbs and abstract nouns convey more ‘information content’ than concrete nouns? Piantodosi and colleagues replied with additional corpora analyses and a resounding “yes”, arguing that verbs and abstract nouns do indeed convey more information content than concrete nouns [104]. The psycholinguistic manifestation of this conclusion remains unclear; however, the optimization of word length for information content does present an alternative hypothesis to the perspective we have advanced here.

In the experiments we reported here, participants showed moderating effects of word form on via semantic judgments of nonwords and real words in a series of offline tasks. In a task more closely approximating ecological language use–single word naming–participants also showed evidence for this effect. Although each of our experiments involved different task demands, participants consistently behaved in non-arbitrary ways in associating specific aspects of word form with word concreteness. The most systematic behavior was evident when making semantic judgments of nonwords (Experiment 1) and in making speeded concreteness judgments for aurally presented real words varied in derivational complexity (Experiment 3). We interpreted these findings in favor of a mechanistic account of statistical learning, consistent with previous work that has found similar effects [60]. However, there exist a number of alternative explanations and methodological considerations that must also be considered. Two consistent criticisms of offline experimental tasks are that they lack ecological validity, and that they lend themselves to potential strategy use. For example, it is possible that participants in the nonword judgment experiment (Experiment 1) used wordlikeness to guide their concrete/abstract judgments. Although we statistically assessed this possibility and attempted to control for a variety of other psycholinguistic variables (e.g., familiarity, lexical density), it is clear that potential confounds persist [105].

### Concluding Remarks

The ability to take advantage of statistical regularities in language can increase the accuracy and efficiency of lexical processing, and is a general principle that has now been observed to operate in several domains of language. Here we have proposed a mechanism for a new form of statistical learning whereby listeners exploit sound structure to speed lexical access for abstract and concrete nouns. This is evidenced by (1) Listeners’ use of word form cues to inform semantic judgments about both nonwords and real words, and (2) Readers’ use of word form cues to facilitate overt naming. Together these results provide converging evidence for the hypothesis that listeners map sound to meaning through a non-arbitrary process using prior knowledge about statistical regularities in the surface forms of words.
